# Surface charges and optical characteristic of colloidal cubic SiC nanocrystals

**DOI:** 10.1186/1556-276X-6-454

**Published:** 2011-07-15

**Authors:** Yong Li, Changxin Chen, Jiang-Tao Li, Yun Yang, Zhi-Ming Lin

**Affiliations:** 1 Technical Institute of Physics and Chemistry, Chinese Academy of Science, Beijing 100190, PR China; 2 Graduate School of the Chinese Academy of Science, Beijing 100039, PR China; 3 National Key Laboratory of Nano/Micro Fabrication Technology, Key Laboratory for Thin Film and Microfabrication of the Ministry of Education, Research Institute of Micro/Nano Science and Technology, Shanghai Jiao Tong University, Shanghai 200240, PR China; 4 P.O. Box 7220, Beijing 100072, PR China

**Keywords:** cubic SiC nanocrystals, surface charges, photoluminescence, quantum confinement

## Abstract

Colloidal cubic silicon carbide (SiC) nanocrystals with an average diameter of 4.4 nm have been fabricated by anisotropic wet chemical etching of microsized cubic SiC powder. Fourier transform infrared spectra show that these cubic SiC nanocrystals contain carboxylic acid, SiH, CH, and CH_x _groups. UV/Vis absorption and photoluminescence (PL) spectroscopy clearly indicate that water and ethanol colloidal suspensions of the as-fabricated colloidal samples exhibit strong and above band gap blue and blue-green emissions. The cubic SiC nanocrystals show different surface charges in water and ethanol solutions due to the interaction of water molecules with polar Si-terminated surfaces of cubic SiC nanocrystals. The results explain the distinctive optical characteristics of colloidal cubic SiC nanocrystals in water and ethanol, and reveal that quantum confinement and surface charges play a great role in determining the optical characteristics of colloidal cubic SiC nanocrystals.

## Introduction

Silicon carbide (SiC) is an important wide band gap semiconductor with superior properties, such as excellent thermal conductivity, high breakdown field strength, and excellent physical and chemical stability [1-4]. It has been found to have applications in many harsh conditions, including high temperature, high power, and high frequency [5] For instance, SiC has been commercially applied for optoelectronic devices, such as photodiodes and light-emitting diodes which emit throughout the visible spectrum into ultraviolet [6]. Its peculiar electronic properties make it a promising material for next-generation electronic devices. It is known that the miniaturization of devices is an irresistible trend for both industrial manufacture and academic research. Furthermore, design and fabrication at the nanoscale would lead to development of the novel materials and devices with target properties [7]. Recent advances in the preparation of SiC nanocrystals have paved the way for wider uses in microelectronic devices and biological labels [8]. Over the past few years, there have been increasing reports on the synthesis of SiC nanocrystals due to their potential applications as nanoscale light emitters [9]. The large band gaps (2.3, 3.0, and 3.2 eV for the main polytypes, 3C-, 6H-, and 4H-SiC) of SiC render the nanocrystals a good candidate as blue and ultraviolet light emitters [10] when quantum confinement takes place. Hence, they could be good candidates for labels of biological molecules and will be superior to Si nanocrystals for its high chemical stability and good biocompatibility. Bulk SiC shows weak emissions at room temperature because of its indirect band gap. The emission intensity can be significantly enhanced when the crystallite size diminishes to several or tens of nanometers. The reason for the above phenomenon is thought to be depressed nonradiative recombination in the confined clusters [11]. Despite the excellent luminescent and electronic properties, the fabrication of SiC nanocrystals has been hindered by its chemical inertness. It is still a great challenge to prepare bulk quantities of SiC nanocrystals with sizes smaller than 10 nm. Up to now, many methods have been developed to fabricate luminescent SiC nanocrystals, such as electrochemical etching of bulk SiC (mostly cubic or hexagonal SiC) [12,13], C ion implantation into bulk Si followed by etching [14], C_60 _introduction into porous silicon followed by annealing [15], chemical etching of microscale cubic SiC powder, and subsequent ultrasonic vibration [16], and so on. Among these methods, catalyzed electrochemical etching of polycrystalline SiC wafers in a hydrofluoric acid (HF)/ethanol solution is most intensively investigated [17,18]. Although hexagonal or cubic SiC nanocrystals with diameters ranging from 1 to 6 nm were achieved, the yield of SiC nanocrystals is limited by the surface area of expensive polycrystalline SiC wafers. Wet electroless chemical etching is a desirable substitute for electrochemical etching because it is relatively inexpensive, can be done with simple equipment, and has large-scale production. Recently, Zhu and coworkers have reported the synthesis of cubic SiC nanocrystals by wet electroless etching of microscale cubic SiC powder in a HNO_3_/HF solution and experimentally observed intense violet-blue photoluminescence (PL) from colloidal cubic SiC nanocrystals [16]. However, the surface chemistry of etched cubic SiC powder from which cubic SiC nanocrystals derived and the influence of surface terminations of cubic SiC nanocrystals in different surrounding environments on its optical properties still need to be investigated in detail.

In this paper, we report successful fabrication of colloidal cubic SiC nanocrystals by anisotropic wet chemical etching of microsized cubic SiC powder. The obtained colloidal cubic SiC nanocrystals are about 4.4 nm in diameter and exhibit significant quantum confinement with strong blue and blue-green emissions. The surface charges and optical characteristic of the as-prepared colloidal cubic SiC nanocrystals are investigated, and the possible reasons are discussed.

## Materials and methods

The cubic SiC powders which were fabricated by a combustion synthesis method using powders of silicon (99.9% pure, 325 mesh, General Research Institute for Nonferrous Metals, Beijing, China) and carbon black (Tian Hao Carbon Black Corporation, Tianjin, China) as starting materials in our lab, and the fabrication process has been described previously [19]. The particle size of the cubic SiC powders determined by laser scattering method is 0.1 μm to approximately 0.3 μm. The cubic SiC powders were etched in a mixture (3:1) of 40% HF and 65% nitric acid (HNO_3_) solutions at 80°C for 3 h. After etching, cubic SiC powders were subsequently washed with deionized water several times and dried at 75°C for several hours. The dried etched cubic SiC powders were then mechanically ground in an agate mortar for 1 h to obtain numerous separated nanocrystals from the etched porous powder. The ground powder was then dispersed in deionized water and ethanol by ultrasonic vibration for 30 min, respectively. The formed suspension was centrifuged at 2,700 × *g *for 1 h. The top part of the suspension was collected, which contains uniformly dispersed cubic SiC nanocrystals. Several measuring and analytical techniques were employed to characterize the as-prepared samples. Transmission electron microscopy (TEM) studies were performed with a JEM-2100 (2,100-kv) microscope (JEOL Ltd., Tokyo, Japan). TEM samples were prepared by dropping a droplet of the cubic SiC nanocrystals in ethanol on a carbon-coated Cu grid. The infrared transmission spectra (FTIR) were acquired using an Excalibur 3100 Fourier transform infrared spectrometer (Varian, Walnut Creek, CA, USA). The UV/Visible absorption spectra of the colloidal cubic SiC nanocrystals were obtained on a Cary 5000 spectrometer (Agilent Technologies Co. Ltd., Beijing, China) over the wavelength range of 200-800 nm by putting the nanoparticle-contained suspension solution in a quartz cell. The PL spectral measurements of the samples were performed in a Hitachi F-4500 fluorescence spectrophotometer with a Xe lamp. All measurements were performed at room temperature.

## Results and discussions

**Figure 1:**
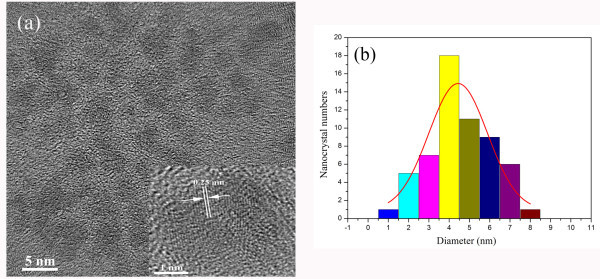
**TEM image of the spherical-like cubic SiC nanocrystals**. **(a) **High-resolution TEM image of 3C-SiC nanocrystals. Insert: magnified image of a single nanocrystal about 4 nm in size. **(b) **Size distribution of 3C-SiC nanocrystals with the most probable size of 4.4 nm obtained by Gaussian fitting.

Figure 1a shows a TEM image of the spherical-like cubic SiC nanocrystals with dimensions smaller than 5 nm dispersed on a carbon-coated Cu grid. As one can see, the cubic SiC nanocrystals are highly crystalline and the lattice fringes can be seen, which correspond to the (111), (200), and (220) planes of cubic SiC, respectively. The insert is the magnified image of a single nanocrystal about 4 nm in diameter, and the lattice fringe space is 0.25 nm, which corresponds to (111) plane of bulk cubic SiC. The size distribution of the cubic SiC nanocrystals obtained from the analysis of the TEM images is presented in Figure 1b. A Gaussian fitting indicates that the maximal probability for nanocrystals' diameter is 4.4 nm. The similar etching process for cubic SiC has been investigated, and the suggested reaction could be expressed as follows [16,18]:(1)

HNO_3 _acts as the oxidizing agent, and the superfluous HF removes the produced silicon oxide. The etching process was not uniform and very selective to some parts of the particles, resulting in the formation of interconnected nanostructure network. It has been reported that there was a large difference in etching rates between different polytypes of SiC [20]. For example, cubic SiC could easily be etched by HNO_3 _and HF, while hexagonal SiC is very stable in such a mixture. The SiC powders prepared by a combustion synthesis method contain a fair concentration of stacking faults due to the nonequilibrium reaction process. The large amount of stacking faults can be verified by the X-ray diffraction profiles [21] and high-resolution electron microscopy observations [22]. Zhang *et al. *[23] have revealed that the stacking faults in the cubic SiC nanorods are threefold in nature, and the region near the threefold stacking faults resemble the structure of hexagonal SiC. Therefore, the structure of the powders may be viewed as a cubic SiC matrix containing nanoscaled hexagonal SiC regions. Different etching rates of hexagonal SiC as compared with the bulk cubic SiC may explain the preferential etching of cubic SiC powders.

**Figure 2:**
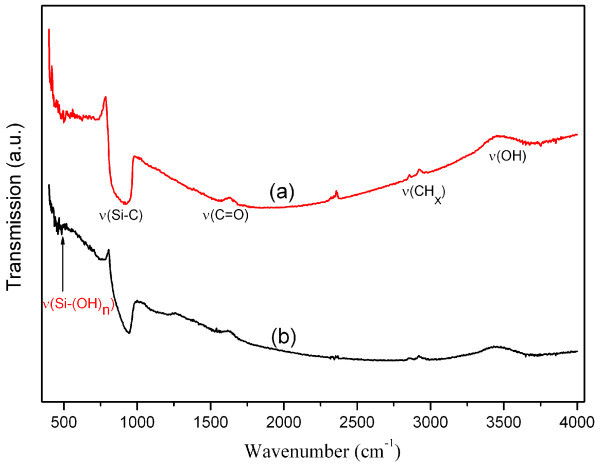
**FTIR spectra of cubic SiC powder**. **(a) **As-prepared and **(b) **etched 3C-SiC powder.

The surface features on etched SiC can influence the solubility, electronic structure, and optical characteristic of SiC nanocrystals [24]. The FTIR spectra of the as-prepared and etched cubic SiC powder are shown in Figure 2. The intense absorption band in 700 to approximately 1,000 cm^-1 ^can be indexed as Si-C characteristic stretching mode of SiC (972 cm^-1^) [25]. The peak position of the etched sample (944 cm^-1^) shows a downshift with respect to that of the as-prepared sample (923 cm^-1^). The downshift may be ascribed to the phonon confinement induced by embedded SiC nanocrystals in the etched sample [26]. The peak at 478 cm^-1 ^is assigned to Si-(OH)_n _vibrations [24]. After chemical etching, this peak disappears. There is a weak peaks *v*(C = O) band at 1,638 cm^-1 ^in the spectrum of etched sample, and this indicates the oxidization and anisotropic chemical etching process on the surface of cubic SiC powders. The bands at 2,989 and 2,943 cm^-1 ^reveal the presence of different aliphatic CH_x _groups in the etched sample. The *v*(OH) band centered at about 3,500 cm^-1 ^corresponds to the absorbed water molecules. Our FTIR results are in agreement with former reports for electrochemical etching cubic SiC [27].

**Figure 3:**
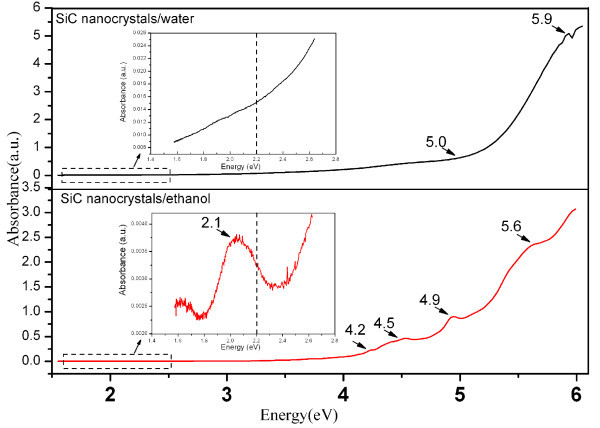
**Absorption spectra of the colloidal cubic SiC nanocrystals in water and ethanol**. The insert shows the enlarged absorption spectra, taken from the dashed line square area.

Previous work has revealed that the UV/Vis absorption spectrum contains much information about the excitation dynamics and the energy levels in the SiC nanocrystals [28]. Figure 3 shows the UV/Vis spectrum of cubic SiC nanocrystals in the water and ethanol suspensions. Below the band gap of bulk cubic SiC (2.2 eV), there is a soft absorption edge for the colloidal cubic SiC nanocrystals in water and an absorption peak at 2.1 eV in ethanol, as shown in the inset of Figure 3. Above the band gap, two suspensions show a strong continuous increase. Pure water and ethanol exhibit no absorption in this range; hence, the light absorption in Figure 3 results from the colloidal SiC nanocrystals. When the incident photon energy is lower than the band gap, colloidal cubic SiC nanocrystals absorb light energy via band tail states, and the above band gap absorption is via band-band transitions in different nanocrystals [9,26]. The colloidal cubic SiC nanocrystals in water show much higher absorption intensity than that in ethanol. The FTIR results indicate that the etched cubic SiC powders posse oxygen-terminated polar surfaces. The oxygen-terminated cubic colloidal SiC nanocrystals have a higher solubility in water, as a result, high absorption intensity is obtained. The cubic SiC nanocrystals in water show a smoother absorption spectrum with the first peak at 5.9 eV. The absorption spectrum show a sharp rise when the incident photo energy is larger than 5 eV. According to the quantum confinement model, the electronic band gap of the nanocrystals increases when its diameters decrease, and the sharp rise in the absorption spectrum arises from a growing number of cubic SiC nanocrystals with band gaps lower than 5 eV. The cubic SiC nanocrystals in ethanol exhibit a step-like absorption spectrum characteristic. There are several sharper absorption peaks (2.1, 4.2, 4.5, 4.9, 5.6 eV) with increasing photo energies. These absorption peaks correspond to the confined energy levels of ultrasmall cubic SiC nanocrystals [29] in ethanol.

**Figure 4:**
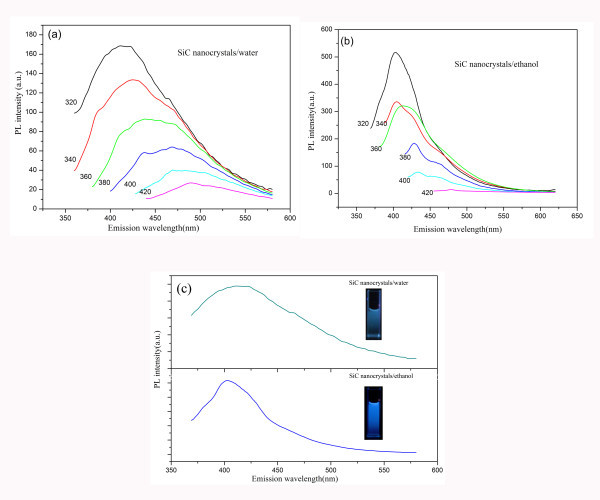
**PL emission spectra of the colloidal cubic SiC nanocrystals**. Room temperature PL spectra of 3C-SiC nanocrystals dispersed in **(a) **ethanol and **(b) **water. **(c) **The PL emission spectra of colloidal cubic SiC nanocrystals in water and ethanol solutions at the excitation wavelength of 320 nm (the insert is two emission photos taken using a Canon digital camera at the same excitation wavelength).

Figure 4a, b depicts the typical PL emission spectra of the colloidal cubic SiC nanocrystals in water and ethanol, which exhibit prominent above band gap blue and blue-green emissions at different excitation wavelengths. As the excitation wavelength increases, the emission peak shows a monotonic red shift. Figure 4c shows the PL emission spectra of colloidal cubic SiC nanocrystals in water and ethanol solutions at the excitation wavelength of 320 nm. As we can see, the full width at half maximum (FWHM) of PL spectra are 127 and 67 nm, respectively. The observed phenomenon can be explained by the solubility of the cubic SiC nanocrystals in water and ethanol. The number of cubic SiC nanocrystals steadily dispersed in ethanol is smaller than that in water, which leads to a narrower FWHM. The insert of Figure 4c is two emission photos taken using a Canon digital camera at the same excitation wavelength. The emission spots are seen to be blue and blue-green, respectively. These results are in good agreement with the previous reports [30].

To theoretically understand the origin of above band gap emissions, several theoretical models have been proposed to deal with the band gap widening of cubic SiC nanocrystals due to quantum confinement and surface reconstruction. The recent *ab initio *theoretical studies reported by Reboredo *et al. *[31] have predicted that silicon carbide band gap is sensitive to interfacial chemical environment. For nanocrystals smaller than 3 nm, the band gap is affected by surface termination through modifying a physical expansion of the lattice [32]. Using the effective-mass approximation, the energy gap *E*_g_* between valence and conduction bands will be widened with the decreasing crystal diameters according to the following equations [18,33,34]:(2)

where *μ *is the reduced mass of the exciton, *μ *= *m*_e_*m*_h_/(*m*_e _+ *m*_h_), *m*_e _= 0.394*m*_0_, *m*_h _= 0.387*m*_0_, *E*_g _= 2.25 eV. *ε *is the high frequency dielectric constant of cubic SiC (*ε *≅10), *ε*_0 _= 8.854 × 10^-12 ^F/m, and *r *is the radius of the nanocrystals. For the cubic SiC nanocrystals with 4.4 nm, we can estimate that the band gap will be widened to 2.51 eV, which is in agreement with the emission peak located at approximately 490 nm.

The surface charges of cubic SiC nanocrystals will form new electronic states in the band gap, and this will influence the PL energy levels. It is reported that the radiative recombination takes place in the surface/defect states. On the surface of cubic SiC nanocrystals, a large number of C and Si terminations will be formed after anisotropic chemical etching and ultrasonic treatment. In the water suspension, the water molecules will be dissociated into -OH and -H groups, which will bond to C and Si terminations. In room temperature, -OH groups only react with Si terminations [32]. The interaction between water molecules and bulk cubic SiC (001) Si-terminated surface leads to dissociation of water molecules accompanied by changes in both its structural and electronic properties. The -OH and -H groups bond mainly on the Si-terminated cubic SiC nanocrystals. Cicero and coworkers [35] have investigated the interaction of water molecules with polar Si-terminated surfaces of cubic SiC by means of *ab initio *molecular dynamics simulations.

According to their results, the dissociation process occurs through a mechanism of proton exchange among the water molecules close to the surface of SiC, and it can be summarized as follows:(3)(4)

**Figure 5:**
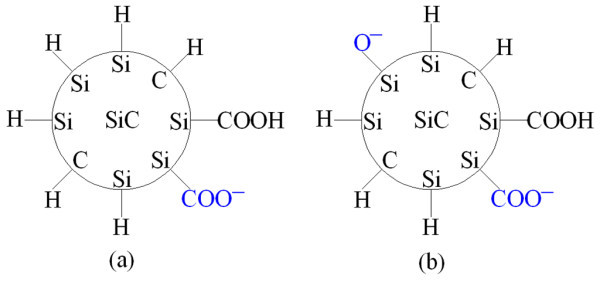
**Surface chemistry of 3C-SiC nanocrystals**. Surface chemistry of 3C-SiC nanocrystals dispersed in **(a) **ethanol and **(b) **water.

The surface acts as the electron acceptor in the process (3) and hole acceptor in process (4). In the ethanol suspension, only -H groups are dissociated. Therefore, only C-H and Si-H groups are formed on the surface of colloidal cubic SiC nanocrystals. The surface charges of cubic SiC nanocrystals dispersed in ethanol and water suspensions are schematically represented in Figure 5. As reported by Wu *et al. *[30], the quantum confinement and Si-OH surface terminations may be the origin of the blue-green PL emission of water suspension.

After storage in air for more than 3 months, the colloidal cubic SiC nanocrystals were found to be uniformly dispersed and not show any appreciable PL spectral shifts or intensity loss. Given their high chemical stability, small size, good biocompatibility, and simple fabrication method, colloidal cubic SiC nanocrystals could find applications in biological labeling as well as in optoelectronic applications.

## Conclusions

In summary, nearly monodispersed colloidal cubic SiC nanocrystals with an average diameter of 4.4 nm have been fabricated via anisotropic wet chemical etching microsized cubic SiC powder. Carboxylic acid, SiH, CH, and CH_x _groups are observed on the etched cubic SiC powder by FTIR spectra. The as-fabricated cubic SiC nanocrystals in water and ethanol exhibit strong, above band gap blue and blue-green PL emissions. Our results reveal that quantum confinement and surface charges in water and ethanol suspensions play a great role in determining the optical characteristic of colloidal cubic SiC nanocrystals. Because of the good biocompatibility, high chemical stability and simple fabrication method, we believe that our results will contribute to the current endeavors aimed at building blocks of nanostructured devices as violet light sources and developing new biological probes in life science.

## Abbreviations

FTIR: fourier transform infrared; FWHM: full width at half maximum; PL: photoluminescence; SiC: Silicon carbide; TEM: transmission electron microscopy

## Competing interests

The authors declare that they have no competing interests.

## Authors' contributions

YL conceived the study and drafted the manuscript; CC conducted the preparation of samples, TEM characterization and analysis of various spectrographic data; J-TL assisted in the design of the study; YY carried out UV absorption analysis; Z-ML performed the FTIR analysis.
